# Heart Rate and Arterial Pressure Changes during Whole-Body Deep Hypothermia

**DOI:** 10.1155/2013/140213

**Published:** 2013-04-11

**Authors:** Giacomo Cavallaro, Luca Filippi, Genny Raffaeli, Gloria Cristofori, Federico Schena, Elisa Agazzani, Ilaria Amodeo, Alice Griggio, Simona Boccacci, Patrizio Fiorini, Fabio Mosca

**Affiliations:** ^1^NICU, Fondazione IRCCS Cà Granda, Ospedale Maggiore Policlinico, Università degli Studi di Milano, Via Della Commenda 12, 20122 Milan, Italy; ^2^NICU, Medical Surgical Feto-Neonatal Department, “A. Meyer” University Children's Hospital, Viale G. Pieraccini, 24, 50139 Florence, Italy; ^3^Neonatal Intensive Care Unit, “Carlo Poma” Hospital, Mantova, Italy

## Abstract

Whole-body deep hypothermia (DH) could be a new therapeutic strategy for asphyxiated newborn. This retrospective study describes how DH modified the heart rate and arterial blood pressure if compared to mild hypothermia (MH). Fourteen in DH and 17 in MH were cooled within the first six hours of life and for the following 72 hours. Hypothermia criteria were gestational age ≥36 weeks; birth weight ≥1800 g; clinical signs of moderate/severe hypoxic-ischemic encephalopathy. Rewarming was obtained in the following 6–12 hours (0.5°C/h) after cooling. Heart rates were the same between the two groups; there was statistically significant difference at the beginning of hypothermia and during rewarming. Three babies in the DH group and 2 in the MH group showed HR < 80 bpm and QTc > 520 ms. Infant submitted to deep hypothermia had not bradycardia or Qtc elongation before cooling and after rewarming. Blood pressure was significantly lower in DH compared to MH during the cooling, and peculiar was the hypotension during rewarming in DH group. *Conclusion*. The deeper hypothermia is a safe and feasible, only if it is performed by a well-trained team. DH should only be associated with a clinical trial and prospective randomized trials to validate its use.

## 1. Introduction

Hypoxic-ischemic encephalopathy (HIE) is a disease that induces death and severe neurological damage in newborns [1]. Hypothermia is now the first effective neuroprotective intervention for newborns that are critically ill following an asphyxial insult. Hypothermia showed a decrease in death or disability at 18 months [2–5]. Parkins et al. firstly introduced hypothermia as a neuroprotective method, but the side effects were higher, so the practice had been abandoned [6]. Meta-analysis about eight largest RCTs had confirmed that cooling reduced mortality without increasing handicap in survivors [7]. Two meta-analyses have confirmed that hypothermia was associated with a lower risk of death or moderate/severe neurodevelopmental disorder in childhood, but with an increased risk of arrhythmia and thrombocytopenia [8, 9]. Perlman suggested that deeper or more prolonged cooling could be able to treat more severe neonatal asphyxia [2]. The early studies of deep hypothermia (20–27°C) have found some neurological benefits, but the side effects dramatically increased below 32°C and passed all potential clinical benefits [10]. Shivering and cardiac arrhythmias were the most pronounced of these side effects [11, 12]. Cardiac arrhythmias, hypotension, hemoconcentration, sinus bradycardia, and peripheral vasoconstriction are common adverse events in neonatal hypothermia [13–15]. Safety and respiratory function during deep hypothermia (DH) in asphyxiated newborn were previously reported [16, 17]. Retrospective analysis of circulatory parameters is part of a prospective study designed to investigate the safety of the topiramate [18, 19]. The objective of this retrospective analysis was to investigate how heart rate and systemic arterial pressure were modified by DH (core temperature 30–33°C). Results were compared to a group of term infants with hypoxic-ischemic encephalopathy (HIE) treated with milder levels of hypothermia (MH) (core temperature 33-34°C).

## 2. Methods

Asphyxiated newborns included in the analysis were those treated with whole-body DH or MH within the first 6 hours of life and if the two following criteria were fulfilled: (1) gestational age ≥36 weeks and birth weight ≥1.800 g, with at least one of the following: (a) Apgar score ≤5 at 10 minutes; (b) endotracheal intubation or mask ventilation for more than 10 minutes after birth; (c) acidosis (pH ≤ 7.0 and/or basic excess ≥−16 mmol/L in umbilical cord blood or arterial, venous, or capillary blood) within 60 minutes from birth; (2) moderate to severe encephalopathy consisting of altered state of consciousness (irritability, lethargy, stupor, or coma) and ≥1 of the following signs: (a) hypotonia, (b) abnormal reflexes, including oculomotor or pupillary abnormalities, (c) absent or weak suctioning, and (d) clinical seizures [3, 10]. Between November 2004 and May 2010, 78 asphyxiated newborns with HIE were retrospectively compared: 37 patients were treated with DH and 41 with MH. Four patients in each group were excluded because they were cooled with ice packs. Nineteen patients in DH and 20 patients in MH were excluded because they received inotropic supply. Fourteen patients in DH and 17 in MH were analyzed.

Patients were cooled by two different cooling blankets, NIG 2 (Iemmi Medical Srl.; Mantova, Italy) and Blanketrol III (Hyper-Hypothermia System, Cincinnati Sub-Zero, Cincinnati, OH, USA). Monitoring was performed by a rectal and esophageal probe. Skin temperature was monitored on the abdominal wall with a skin probe by the radiant warmer thermal sensor or a temperature-monitoring unit (Mon-a-therm; Mallinckrodt Medical, St. Louis, MO, USA). The target of the core temperature was 31°C and 33.5°C for DH and MH, respectively. Cooling was maintained between the stated targets for 72 h, and rewarming to 36.5–37°C was gradually obtained over the following 6–12 h (0.5°C/h).

Arterial blood gas, coagulation, and biochemistry were daily checked and temperature corrected. Chest X rays and echocardiography (Acuson Sequoia C512, Siemens, Germany) were performed at the admission. The scans were performed by 2 experienced operators. Respiratory rate, heart rate (HR), blood pressure (BP), and oxygen saturation were continuously monitored during hypothermia and rewarming. BP was measured by using standard oscillometric methods. Invasive blood pressure monitoring was not performed routinely. Only oscillometric blood pressure values were selected for analysis to reduce the bias. Standard electrocardiogram was obtained when bradycardia (HR of <80 bpm) or arrhythmia (extrasystoles, bundle branch block) was observed. Hypotension, defined as mean arterial blood pressure (MAP) <40 mm Hg, was, firstly, treated with single or repeated normal saline boluses (10–20 mL/kg) and subsequently by dopamine, while dobutamine was infused when the reduction of myocardial contractility was found. Dopamine or dobutamine was started at an initial dose of 5 ug/kg/min and was increased by 5 ug/kg/min up to a maximum dose of 15 ug/kg/min. Fluid intake was started at 60 to 70 mL/kg and increased by 10 to 20 mL/kg each day, based on changes in body weight and serum electrolyte levels. Clinical seizures were treated with phenobarbital (loading dose, 20 mg/kg; followed by 1.5–2.5 mg/kg every 12 hours). Oral topiramate was administered as neuroprotective drug at starting time of hypothermia, once a day for 3 days [18, 19]. Inhaled nitric oxide was started when PPHN was echographically demonstrated. 

Brain magnetic resonance imaging (MRI) was planned within the first week of life. Brain lesions were classified as isolated lesions of white matter, basal ganglia, and thalamus with or without involvement of posterior limb of the internal capsule, cortex, or various combinations of the earlier lesions [20].

## 3. Statistical Analyses

Results were shown as mean ± SD or percentage. Statistical analyses were performed using Student's *t*-test, Mann-Whitney test, and ANOVA; categorical variables were analyzed using Fisher exact test or *χ* ^2^ analyses. A paired *t*-test was used to determine the effect of a single treatment. *P* value of ≤0.05 was considered significant.

## 4. Results

**Figure 1:**
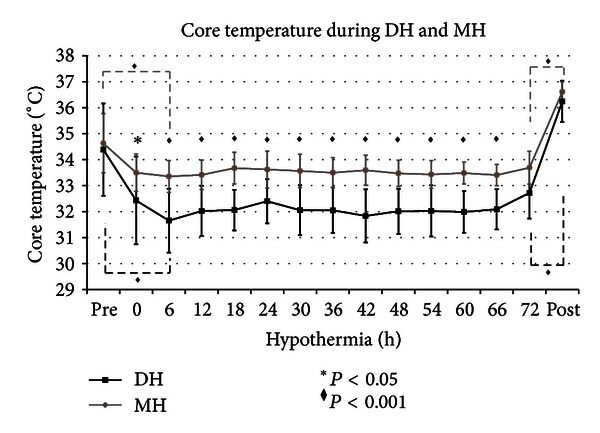
Core temperature during DH and MH (mean; standard deviation). The black and grey solid lines represent the core temperature during DH and MH. The black asterisk (∗) represents the statistical significance with *P* < 0.05. The black diamond (*◆*) represents the statistical significance with *P* < 0.001. Core temperatures were the same between the two groups at starting hypothermic time; then the mean core temperature was 31°C and 33.5°C in DH and MH, respectively. DH and MH had an analogous trend during cooling and rewarming. There was a statistical significant difference (*P* < 0.001), from 6 to 72 hours, between the two trends during cooling. There was statistical significance between prehypothermic and hypothermic temperature and hypothermic and postrewarming temperature (*P* < 0.001).

**Figure 2:**
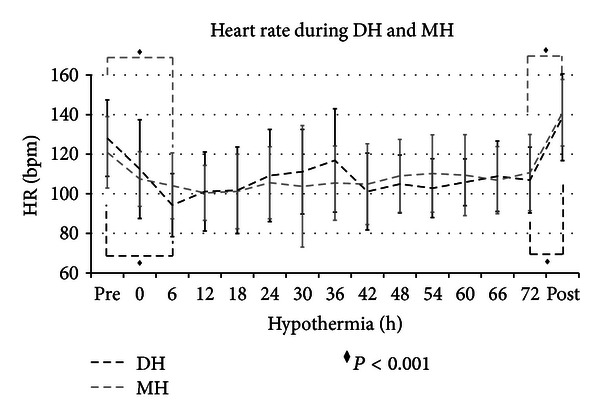
Heart rate during DH and MH (mean; standard deviation). The black dashed line represents the heart rate (HR) of newborns in deep hypothermia (DH). The grey dashed line represents the HR of newborns in mild hypothermia (MH). The black diamond (*◆*) represents the statistical significance with *P* < 0.001. HR was very comparable between the two groups at starting hypothermic time and during hypothermia. There was statistical significance between prehypothermic and hypothermic HR and hypothermic and postrewarming HR (*P* < 0.001).

**Figure 3:**
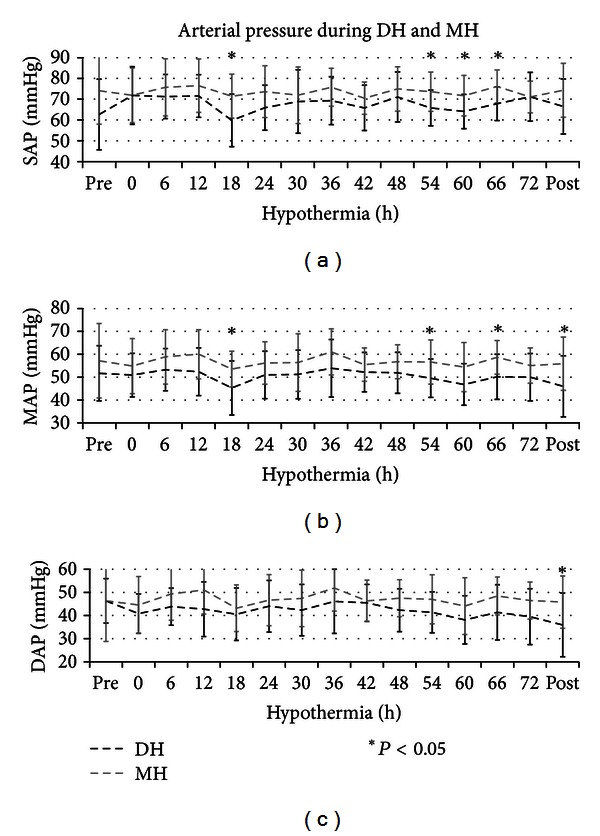
Change in arterial pressure during DH and MH (mean; standard deviation). The systolic (SAP), mean (MAP), and diastolic arterial pressure (DAP) was represented with black and grey dashed lines in patients during deep hypothermia (DH) and mild hypothermia (MH), respectively. The black asterisk (∗) represents the statistical significance with *P* < 0.05.

Newborns reported in three earlier study were included [17–19]. Seventy-eight asphyxiated newborns were retrospectively compared from November 2004 to May 2010: 37 patients were treated with DH, and 41 with MH. Four patients in both groups were excluded because they were cooled with ice packs. Nineteen patients in DH and 20 patients in MH were excluded because they received inotropic supply. Fourteen patients in DH and 17 in MH were analyzed, and there was not a statistically significant difference between the two groups (*P* = 0.855). Perinatal and basic characteristics of the infants were not significantly different (Table 1). Core temperatures were the same between the two groups at hypothermic starting time. During treatment, the core temperature was, respectively, maintained at 31°C and 33.5°C in DH and MH. There was a statistical significant difference (*P* < 0.001) from 6 to 72 hours between the two groups (Figure 1). At starting cooling, mean HR was 110 bpm and 111 bpm during DH and MH, whereas it was reduced at 97 bpm and 105 bpm during DH and MH (Figure 2). Three babies in DH (20%) and 2 in MH (11.7%) showed a sinus bradycardia (HR < 80 bpm), with a significant increase in QTc (>520 ms) during hypothermia (*P* = 0.326). Newborns did not have bradycardia before cooling and after rewarming. Heart rate decreased significantly by a mean of 11 bpm in DH and nine bpm in MH on cooling (Figure 2). Sinus bradycardia was not associated with a decrease of mean arterial pressure (MAP), and arrhythmia has not been reported. Mean systolic arterial pressure was 66 mmHg and mean diastolic blood pressure was 41 mmHg, during DH. During MH, mean systolic blood pressure was 72 mmHg, and mean diastolic blood pressure was 46 mmHg (Figure 3). Blood pressure was significantly lower in DH than MH, and DH group presented a greater decrease in MAP during rewarming compared with MH with several statistical significance. Two newborns in MH and one in DH died, and that occurred 72 hours after hypothermia. Length of hospitalisation was similar in both groups (Table 1).

## 5. Discussion

Cardiovascular changes during cooling have a significant incidence on systemic sequelae in infants with HIE. The experimental data have shown that during mild hypothermia, left ventricular contractility and cardiac output decreased [21–23]. This is the first report that compares the cardiovascular function in treated asphyctic newborns with two different degrees of hypothermia. This report has demonstrated how the MAP changed during DH while HR remained the same changing degrees of cooling. In both treatment groups, target temperature was reached and maintained. Cooling was the same even using two different devices, and statistical difference was significant during hypothermia (Figure 1). Indeed, Hoedemaekers et al. showed that the use of different blankets did not change the induction and maintenance of cooling [24]. 

Thoresen and Whitelaw have documented how HR and MAP changed in asphyxiated newborns during hypothermia and rewarming. In fact, while the MAP increased, HR decreased during the cooling, and the opposite occurred during the rewarming [25]. Already three randomized controlled trials have been reported as a decrease in HR was reversible [3, 4, 13]. In the same way, it was already described as neonates undergoing hypothermia were also exposed to sinus bradycardia and hypotension [7, 12, 26]. Furthermore, QTc elongation is a typical anomaly during hypothermia. Gunn et al. reported that HR of infants submitted to head cooling decreased and, at the same time, increased the QT interval. After rewarming, HR and QT interval came back to normal range [20, 27–30]. 

In this report, HR rapidly decreased during cooling induction and quickly increased during passive rewarming. Sinus bradycardia has been observed in both groups, while a significant increase in QTc developed only in 5 patients. The depth of hypothermia did not appear to affect HR while the induction of cooling and the gradual warming seemed to affect it (Figure 3).

Studies on pressure data are contradictory, because some have reported a pressure decrease, while others have documented an increase in blood pressure during hypothermia [7, 25].

In this report, blood pressures inevitably fall after the beginning of hypothermia in both groups and more in that subjected on deep hypothermia (Figure 3). However, a further decrease in blood pressure had been observed during rewarming only in DH group, as described by Thoresen and Whitelaw [25]. Although the rewarming rate was similar in both groups (0.5°C/h), we assumed that this rate was too elevated for those patients undergoing a more deep cooling. Therefore, based on previous studies, it is to suggest that there may be some benefit to give volume at the time of rewarming to “prime” the circulation [31].

Blood pressure significantly lower in the DH may be explained with inhibition of endogenous catecholamines activity following the further reduction on body temperature below 33°C. Indeed, Chernow et al. measured HR, MAP, and plasma levels of catecholamines during progressive hypothermia to 29°C and then during rewarming to 37°C on baboon. HR, MAP, and plasma levels of epinephrine and norepinephrine increased significantly when the temperature dropped to 33°C. A further reduction in temperature to 29°C corresponded with a reduction in HR, MAP, and catecholamine to prehypothermic levels. Therefore, sympathetic nervous system immediately responds to hypothermia but may be “switched off” at threshold temperature of about 29°C [32].

We also speculated that hypotension was probably also due to hypoxic multiorgan or multisystemic dysfunction (MOD) [33]. It is necessary to remember that MOD as renal, cardiovascular, pulmonary, or hepatic involvement is frequent in postasphyxial infants and is, respectively, present in 70%, 62%, 86%, and 85% of infants with HIE [33].

The adverse effects of deep cooling may be more severe in asphyxiated newborns with multiorgan dysfunction, or multiorgan dysfunction could be amplified by superimposed cold-injury syndrome [34, 35]. In an experimental adult dog model, deep hypothermia (15°C) produced worse cerebral and cardiac outcomes [36], whereas controlled mild hypothermia (34–36°C) provided consistent neuroprotective benefits [37]. The mechanisms of adverse effect of deep hypothermia or “overcooling” on cerebral outcome are not clear but might be related to myocardial dysfunction leading to decrease in cardiac output, systemic hypotension, and compromised cerebral perfusion [22, 36, 38, 39]. Deep hypothermia used in this study did not seem to be as profound as that used on the dogs by Dudgeon et al. [22], but it could itself be responsible to hypotension.

## 6. Conclusion

In conclusion, the present report confirms that patients under DH are also subjected to cardiovascular dysfunction. Indeed, DH patients are subject to an increased risk of hypotension both during and after hypothermia. Small sample size, retrospective data collection, BP recording method, and observational clinical markers of cardiovascular status limit the power of our conclusions. However, deep hypothermia is safe and feasible treatment only if performed by a well-trained team [16, 17]. While moderate hypothermia can now be considered a “standard care” in asphyxiated term infants, DH should only be associated to a clinical trial. New prospective randomized trials are needed to validate its use.

## Figures and Tables

**Table 1:** Basic characteristics of the group. (mean ± SD or *n* and %).

	Group DH *n* = 14/31 (45.1)	Group MH *n* = 17/31 (54.9)	*P* value	95% C.I.
Weight g (SD)	3296 (±659)	3292 (±608)	*P* = 0.982	−299.0–305.7
Gestational age weeks (SD)	39.6 (±1.7)	39.2 (±1.9)	*P* = 0.488	−0.737–1.222
Inborn *n* (%)	5/14 (35.7)	7/17 (23.5)	*P* = 0.738	−0.482–0.726
Male *n* (%)	6/14 (42.8)	12/17 (70.5)	*P* = 0.533	−0.754–0.200
Caucasian race *n* (%)	11/14 (78.5)	13/17 (76.4)	*P* = 0.714	−0.315–0.357
Vaginal delivery *n* (%)	6/14 (42.8)	10/17 (58.8)	*P* = 0.897	−0.606–0.406
Apgar 5′ (SD)	4.8 (±2.0)	4.1 (±2.1)	*P* = 0.419	−0.334–1.607
Umbilical cord pH (SD)	6.881 (±0.1)	6.996 (±0.2)	*P* = 0.066	−0.397–0.079
Umbilical cord paCO_2_ (mmHg) (SD)	90.2 (±34.0)	68.3 (±32.4)	*P* = 0.080	−2.730–46.423
Umbilical cord BE (mmol/L) (SD)	−17.0 (±4.1)	−17.7 (±7.1)	*P* = 0.981	−2.592–7.207
Umbilical cord lactate (mmol/L) (SD)	12.1 (±6.3)	14.1 (±4.6)	*P* = 0.378	−6.694–2.632
Onset of hypothermia h (SD)	3.2 (±1.5)	3.3 (±1.2)	*P* = 0.957	−0.697–0.528
Length of hospitalization (days) (SD)	16.7 (±12.9)	13.1 (±7.2)	*P* = 0.270	−0.201–9.171

DH: deep hypothermia. MH: mild hypothermia. SD: standard deviation. BE: basic excess. h: hours.
